# Spontaneous scapular spine fracture related to rotator cuff pathology: a report of two cases

**DOI:** 10.1007/s11751-012-0135-6

**Published:** 2012-05-19

**Authors:** Diederik Groot, Anouk M. E. Giesberts, Jan B. A. van Mourik

**Affiliations:** 1 Department of Orthopedic Surgery, MCA Hospital Alkmaar, Wilhelminalaan 12, 1815 JD Alkmaar, The Netherlands; 2 Orthopedic Department, Maxima Medisch Centrum, Ds. Th. Fliednerstraat 1, postbus 90052, 5600 PD Eindhoven, The Netherlands

**Keywords:** Spontaneous fracture, Scapula, Cuff arthropathy

## Abstract

Spontaneous fractures of the scapula are rare, especially those involving the scapular spine. There are only a few case reports addressing this topic. Two cases are presented of spontaneous scapular spine fractures in patients with cuff-tear arthropathy. Treatment was conservative, resulting in a stiff shoulder in both patients. The combination of oral steroids and cuff-tear arthropathy seems to have caused a spontaneous scapular spine fracture in these patients. Considering the risk of operative intervention in the elderly patient conservative treatment seems a reasonable alternative.

## Introduction

Scapular fractures are usually caused by severe trauma [1]. Stress fractures of the acromion were found to be related to cuff-tear arthropathy [2]. Spontaneous fractures of the scapular spine were described by Shindle et al. [3] and treated operatively. Osteoporosis is a known risk factor for the development of atraumatic fractures. Two cases of patients with spontaneous scapular spine fractures and aetiological factors involved are discussed. Both patients showed osteopenic bone on X-rays and both patients were treated conservatively, resulting in a stiff shoulder. The overall outcome was reasonable and acceptable in both patients.

## Patients and methods

### Case 1

**Fig. 1:**
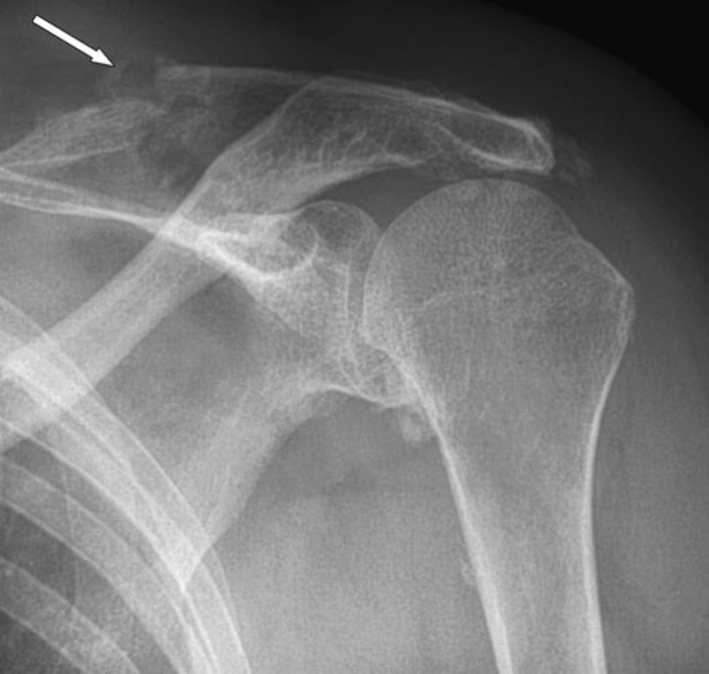
(Patient 1) X-ray of left shoulder showing scapular spine fracture, two months after initial symptoms (*white arrow*)

**Fig. 2:**
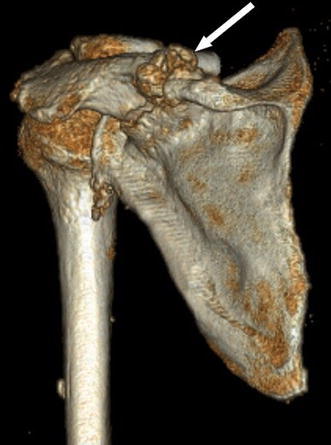
(Patient 1) CT reconstruction of left shoulder showing scapular spine fracture (*white arrow*)

A 72-year-old female was seen at the orthopaedic outpatient clinic with pain in her left shoulder that started after hanging the laundry to dry 2 months earlier. Her medical history includes hypertension, diabetes and chronic inflammatory neuropathy. The neuropathy was treated with prednisone, azathioprine and plasmapheresis. Physical examination revealed a haematoma in the upper left arm, tenderness of the scapula on palpation, active flexion of 100°, abduction of 100° with crepitations, external rotation of 45° and loss of abduction strength. An X-ray of the left shoulder showed a fracture of the scapular spine (Fig. 1). CT scan of the shoulder showed no signs of osteolysis and no sign of consolidation of the scapula fracture (Fig. 2). An MRI scan showed a massive rotator cuff tendon lesion, involving the infra and supraspinatus tendon. Treatment consisted of physiotherapy and a subacromial and intra-articular injection with corticosteroids, which had limited effect on pain and function. The fracture of the scapula was treated with ultrasound bone growth stimulation because the X-ray showed some callus formation but no sign of consolidation. Ten months after initial presentation, she returned with recurrent spontaneous severe pain in the left shoulder, haematoma of the upper left arm and breast discharge. Haematologic evaluation showed no bleeding or coagulation disorders. An X-ray of the shoulder showed no consolidation of the fracture. After discussing operative and conservative options, she chose conservative treatment. At her last follow-up, she had developed a stiff shoulder. The shoulder pain is sufficiently treated with analgesics.

### Case 2

**Fig. 3:**
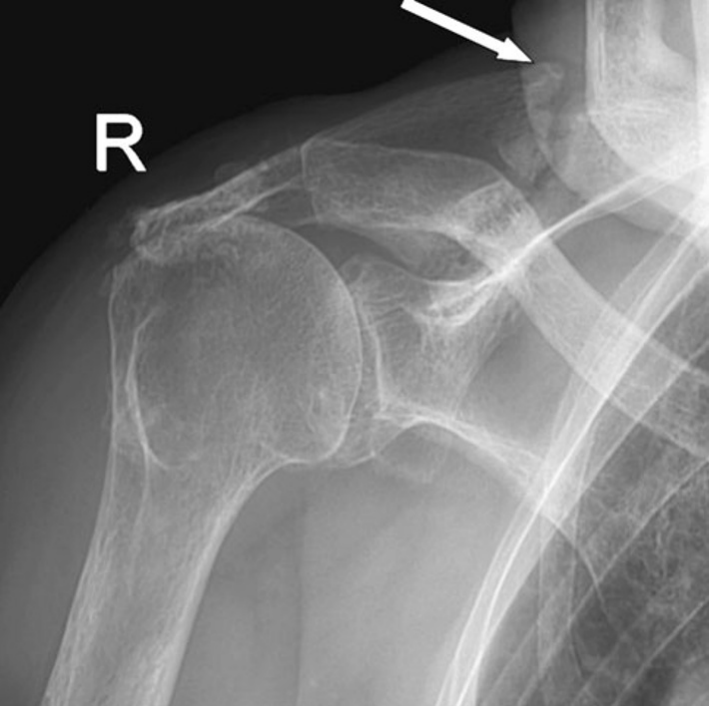
(Patient 2) X-ray of right shoulder showing fracture of the right scapular spine with callus formation but no consolidation 3 months after initial symptoms

**Fig. 4:**
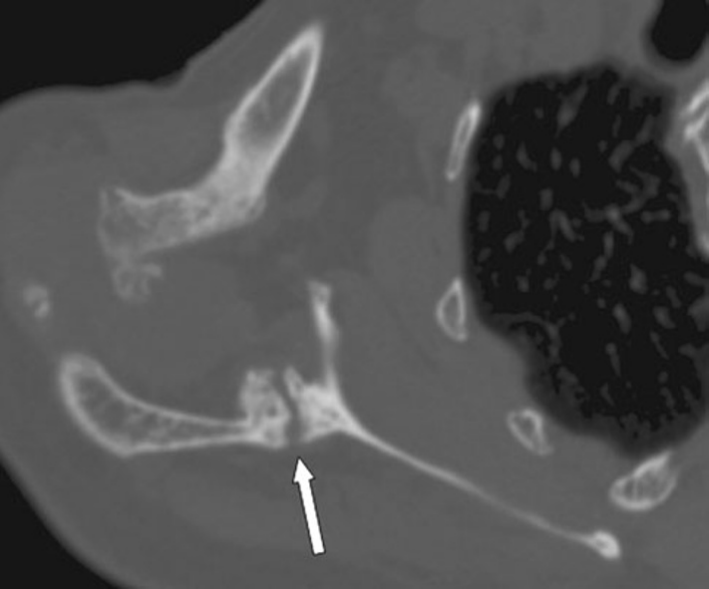
(Patient 2) CT scan of scapular spine fracture, showing callus formation but no consolidation

An 86-year-old female presented with spontaneous progressive pain in her right shoulder that was present for approximately 3 months. Her medical history included hypercholesterolaemia, osteoarthritis of the hip, degenerative spinal scoliosis, COPD, osteoporosis, polymyalgia rheumatica and weight loss of unknown origin. She was treated with prednisone for her polymyalgia rheumatica. Two years earlier, she had had an episode of right shoulder pain after a fall on the right hip and shoulder. An X-ray of the right shoulder at that time showed superior migration of the humeral head but no fracture (Fig. 3). An X-ray and CT scan at time of presentation showed a fracture of the scapular spine with callus but no sign of consolidation (Figs. 3, 4). Treatment consisted of a subacromial injection with corticosteroids because of persistent pain. Ten months after onset, the pain worsened and the upper right arm was increasingly swollen. On physical examination, temperature was normal and there was severe tenderness on palpation over the right scapula. Active glenohumeral abduction was 90° with pain and crepitations, and external rotation was 30°. Clear yellow fluid was aspirated from the glenohumeral joint. Culture of the aspiration was negative. Laboratory results showed an ESR of 27 mm without further abnormalities. After discussing different treatment options, the patient elected conservative treatment. Treatment consisted of circumduction exercise and ultrasound bone growth stimulation. At 3-month follow-up, she had little shoulder function with abduction of 60° and absent external rotation, without pain. An X-ray showed progressive callus formation but no sign of consolidation. The functional impairment was acceptable for the patient.

## Discussion

Fractures of the scapula are usually the result of severe trauma. The cause for scapular fractures without adequate trauma remains unclear. Possible causes found in the literature include stress events with coughing, sport activities such as golf or baseball and work-related activities of the upper extremity [4–7].

A fracture in these cases is the result of stress, direct or indirect, usually caused by repetitive muscle contractions.

Rotator cuff-tear arthropathy can act as a stress riser. Superior migration of the humeral head results in microtrauma of the acromion which in time can lead to a fracture, usually at the base of the acromion [2, 8].

Spontaneous scapular spine fractures are very rare. We only found one report in the literature discussing two similar cases [3].

Shindle describes a possible role of changed biomechanics of the glenohumeral joint. In patients with a rotator cuff lesion, the trapezius muscle and deltoid muscle show increased activity with abduction. These muscles act as a lever on the scapular spine [8]. This stress riser may in time result in a fracture of the spine of the scapula.

In both our cases, rotator cuff-tear arthropathy was present, so the theory proposed by Shindle may be applicable. Furthermore, both patients had a history of oral steroid medication treatment and had signs of osteopenia on the X-rays. We believe that the combination of oral steroids causing osteopenia and cuff-tear arthropathy has caused a spontaneous scapular spine fracture in these patients.

Treatment consisted of physiotherapy, analgesics and ultrasound bone growth stimulation. In both cases, the patients developed a stiff shoulder with acceptable pain, but no consolidation of the fracture had occurred. The possibility of surgical treatment was considered but not accepted by both patients. Possible operative options include internal fixation of the fracture or reversed total shoulder arthroplasty as presented by Shindle et al. These procedures can cause severe complications and are therefore reserved for the more demanding and fit patients. The patients discussed in this manuscript are elderly and have considerable co-morbidity. Both patients had been explained all treatment options but favoured conservative treatment.

## Conclusion

Rotator cuff-tear arthropathy is a risk factor in developing a spontaneous scapular spine fracture. In patients with pain around the scapula who suffer from osteoporosis and have a history of oral steroids treatment, a scapular spine fracture should be considered. Even though the patients ended up with stiff shoulders, the results were reasonable and conservative treatment can be a viable alternative in these elderly patients.
